# Negative regulation by Programmed Death Ligand-1 during drug-specific priming of T-cells and the influence of Programmed Death-1 on effector T-cell function

**DOI:** 10.1186/2045-7022-4-S3-O2

**Published:** 2014-07-18

**Authors:** Andrew Gibson, Monday Ogese, Andrew Sullivan, Eryi Wang, Katy Saide, Paul Whitaker, Daniel Peckham, Lee Faulkner, B Kevin Park, Dean J Naisbitt

**Affiliations:** 1 Department of Molecular and Clinical Pharmacology, University of Liverpool, Liverpool, UK; 2 Regional Adult Cystic Fibrosis Unit, St James's Hospital, UK

## 

PD-1 (Programmed Death-1) has been classified as a marker of T-cell exhaustion, however, several recent studies suggest that most PD-1 high T-cells are highly proliferative effector memory cells that maintain effector function during chronic infection. Furthermore, activation of PD-1 on T-cells is thought to inhibit antigen-specific T-cell priming and regulate T-cell differentiation. Thus, we sought to measure the drug-specific activation of naïve T-cells after perturbation of PD-L1/2/PD-1 binding and investigate whether PD-1 signalling influences the differentiation of T-cells. Naive T-cells were cocultured with monocyte-derived dendritic cells in the presence of a drug (flucloxacillin, nitroso sulfamethoxazole) for a period of 8 days (±PD-L1/2 block), to expand the number of drug-responsive T-cells. The T-cells were then incubated with fresh dendritic cells and the drug. The antigen responsiveness was analyzed using readouts for proliferation, cytokine secretion, and cell phenotype. Cell phenotype was characterised by flow cytometry. T-cell clones were generated following priming and from drug hypersensitive patients to characterize the cytokine signature(s) of antigen specific T-cells and to study whether PD-1 expression/signalling governs the differentiation of T-cells into effector/helper subsets. Priming of naïve CD4+ and CD8+ T-cells against drug antigens was found to be more effective when PD-L1 signaling was blocked. Upon restimulation, T-cells proliferated more vigorously and secreted increased levels of IFN-γ, IL-13 and IL-22, but not IL-17. Naïve T-cells expressed low levels of PD-1; however, CFSE analysis revealed a transient increase during priming. Drug-specific T-cell clones generated through priming and from hypersensitive patients were found to secrete IFN-γ, IL-5 and IL-13. More detailed analysis revealed two different cytokine signatures. Clones secreted either FasL/IL-22 or granzyme B. The FasL/IL-22 secreting clones expressed the skin homing receptors CCR4, CCR10 and CLA and migrated in response to CCL17/CCL27. PD-1 was stably expressed at different levels on clones; however, PD-1 expression did not correlate with the strength of the antigen-specific proliferative response or the secretion of cytokines/cytolytic molecules. This study shows that PD-L1/PD-1 binding negatively regulates the priming of drug-specific T-cells. ELIspot analysis uncovered an antigen-specific FasL/IL-22 secreting T-cell subset with skin homing properties.

